# Success Rate of Endoscopic Sphenopalatine Artery Ligation for the Management of Refractory Posterior Epistaxis Patients in a Tertiary Care Hospital: A Descriptive Cross-sectional Study

**DOI:** 10.31729/jnma.5855

**Published:** 2020-12-31

**Authors:** Meenakshi Basnet, Bibek Ghimire, Akriti Shrestha, Gyan Raj Aryal

**Affiliations:** 1 Department of Otorhinolaryngology and Head and Neck Surgery, Nobel Medical College and Teaching Hospital, Biratnagar, Nepal

**Keywords:** *endoscopy*, *epistaxis*, *Nepal*, *sphenopalatine artery*

## Abstract

**Introduction:**

Epistaxis is a common medical emergency with 5% to 15% of patients admitted for epistaxis will require surgical management as nasal packing has high failure rates. A modern endoscopic technique like Endoscopic Sphenopalatine Artery Ligation has increased in popularity for managing intractable posterior epistaxis. It has less complication and high success rate. The study conducted to estimate the success rate of Endoscopic Sphenopalatine Artery Ligation of refractory posterior epistaxis among admitted patients in a tertiary care hospital.

**Methods:**

This is a descriptive cross-sectional study conducted from June 2019 to June 2020 at the Department of Otorhinolaryngology, Nobel Medical College and Teaching Hospital among the patient with refractory posterior epistaxis with the help of retrospective data. Convenient sampling method was used. These patients underwent endoscopic sphenopalatine artery cauterization for recurrent/intractable posterior epistaxis. Ethical clearance was taken from the Institutional Review Board. Data were analyzed in Statistical Package for the Social Sciences.

**Results:**

Out of the total patient with refractory posterior epistaxis who underwent Endoscopic Sphenopalatine Artery Ligation, the overall success rate was 39 (95.12%). Among them, 25 (60.97%) males and 16 (39.02%) females underwent endoscopic sphenopalatine artery ligation. Twenty (48.78%) of them were unilateral whilst 21 (51.21%) were bilateral disease. About 2 (4.8%) case had re-bleeding within 48 hours which was managed conservatively. Hypertension was found to be the most common comorbid condition followed by diabetes, chronic kidney.

**Conclusions:**

From our study, we conclude that the success rate for Endoscopic Sphenopalatine Artery Ligation in a patient with refractory posterior epistaxis was high.

## INTRODUCTION

Epistaxis is a common emergency condition requiring a sound understanding of nasal vasculature for its management. It has a prevalence of 12%.^1^ Previously, anterior, and posterior nasal packing were an effective treatment for intractable epistaxis. About 5% to 15% of patients requiring admission for epistaxis will require surgical management. Surgical intervention is more likely in the setting of posterior epistaxis, constituting 10% of all cases of epistaxis, due to a higher failure rate of nasal packing (26-52%).^2,3^ The rate of rebleeding is increased to 70% in patients with bleeding disorders.^4^

The management of epistaxis enjoys a wide range of strategies and treatment options. Recent literature advocates an earlier surgical intervention with Endoscopic Sphenopalatine Artery Ligation (ESPAL) due to its simplicity, high success rate, low risks, and cost-effectiveness compared to other treatment modalities. Ligation of the sphenopalatine artery (SPA) as it enters the nose, elevates the success rate.

The objective of the study was to estimate the success rate of ESPAL among admitted patients with refractory posterior epistaxis in a tertiary care center.

## METHODS

This is a descriptive cross-sectional study conducted in the Department of Otorhinolaryngology, Nobel Medical College and Teaching Hospital, Biratnagar, Nepal from June 2019 to June 2020 among intractile posterior epistaxis patient who underwent Endoscopic Sphenopalatine Artery Ligation with the help of retrospective data. Ethical clearance was taken from the Institutional Review Committee of Nobel Medical College. Informed consent was taken from all patients. The patients having posterior/intractable bleeding, not on any anti-coagulants, willing to undergo surgery were included in the study. Those patients who had a bleeding disorder, trauma, nasal mass, having anterior/posterior packing and requiring other artery ligation were excluded from the study. Convenient sampling method was used.

The sample size was calculated by using the formula,

$$\begin{array}{ll} \text{n} & \text{= }{\text{Z}}^{\text{2}}\times \text{p}\times \text{q}/{\text{e}}^{\text{2}}\\ & \text{= }{\left(1.96\right)}^{\text{2}}\times 0.12\times (1-0.12)/{(\text{0.1)}}^{\text{2}}\\ & \text{= }40.56\\ & \approx 41 \end{array}$$

where,

n = sample size
Z = 1.96 at 95% Confidence Interval
p = prevalence, 12%1
q = 1-p
e = margin of error, 10%

About 41 patients with intractable posterior epistaxis underwent ESPAL. All the patients undergoing ESPAL were subjected to routine blood examination. A minimum of 2 pints of blood was arranged after cross-matching. Patients were hemodynamically stabilized prior to surgery. Surgery was performed under general hypotensive anesthesia. Throat packing was done to prevent the swallowing of blood. The nose was packed with neuropathies soaked in 0.05% oxymetazoline were placed in the nasal cavity in the middle and inferior meatus and removed after 5 minutes. Under endoscopic guidance, after careful medial displacement of the middle turbinate, 1 mL of 1% lidocaine hydrochloride with 1:100,000 adrenaline was injected submucosally into the posterior part of the lateral wall of the middle meatus. A vertical 1cm mucoperiosteal incision was made immediately behind the posterior fontanelle, about 1cm anterior to the posterior insertion of the middle turbinate extending from high up in the middle meatus (at the level of the basal lamella) to the inferior turbinate. A posterior dissection was then carried submucosally till crista ethmoidalis was identified. The sphenopalatine artery (SPA) is located just posterior to it. Kerrison's punch is used to properly expose the SPA. It was cauterized with bipolar cautery and cut. The note was made if there was more than 1 branch and ligated after cautery. The flap was replaced, and a small square of Surgicel (Johnson & Johnson, New Brunswick, New Jersey) was applied to maintain its position and avoid minor bleeding. Nasal packing was not done in any patients and was discharged within a day or two. They were followed up manually for 2 months.

The success rate for an ESPAL procedure was defined as no further epistaxis or rebleed within two months of the ESPAL procedure. All the data were collected and entered in Microsoft Excel and was analyzed in Statistical Package for the Social Sciences (SPSS) version 22.

## RESULTS

In our study, the patient with refractory posterior epistaxis who underwent Endoscopic Sphenopalatine Artery Ligation (ESPAL), the overall success rate was 39 (95.12%). There was mild bleeding in 2 (4.87%) cases within 48 hours and were managed conservatively with medical management without nasal packing.

Out of the total of 41 patients, 25 (60.97%) males and 16 (39.02%) females underwent Endoscopic Sphenopalatine Artery Ligation (ESPAL). The age ranged from 14 to 77 years with the mean being 50.59 years (Table 1).

**Table 1:** Sex and age distribution among refractory posterior epistaxis patients who underwent ESPAL (n = 41).

Variables	n (%)
Sex	
Male	25 (60.9l)
Female	16 (39.02)
Age range	
14-25 yrs	1 (2.43)
26-35 yrs	l (1l.08)
36-45 yrs	6 (14.63)
46-55 yrs	10 (24.40)
56-65 yrs	9 (21.95)
>65 yrs	8 (19.51)
Total	41 (100)

**Figure 1:**
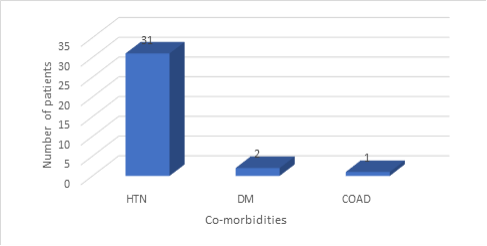
Co-morbidities associated with refractory posterior epistaxis patients who underwent ESPAL.

Co-morbidities were found in 34 (82.92%) patients. Hypertension was found to be the most common comorbid condition amounting to 31 (75.60%) of the cases (Figure 1). Other associated co-morbidities were 2 (4.87%) Diabetes Mellitus (DM), 1 (2.43%) chronic obstructive airway disease (COPD), and 1 (2.43%) hypertension (HTN) with chronic kidney disease (CKD). There were no identifiable co-morbidities in 7 (17.07%) cases.

**Figure 2:**
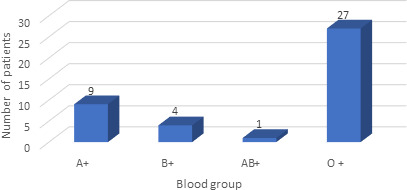
Blood group distribution with refractory posterior epistaxis patients who underwent ESPAL.

There were 20 (48.78%) unilateral nasal bleeding and 21 (51.21%) bilateral nasal bleeding, Thus, twenty out of 41 (48.78%) cases had unilateral whilst 21 (51.21%) had bilateral ESPAL done. There was mild bleeding in 2 (4.87%) cases within 48 hours and were managed conservatively with medical management without nasal packing. In one of the two (2.43%) cases, there was both hypertension and CKD. Maximum patient, 27 (65.85%) undergoing ESPAL had O positive blood group followed by A positive 9 (21.95%) (Figure 2).

About 17 (41.46%) patients consumed alcohol on regular basis. About 5 (12.19%) patients undergoing ESPAL had deviated nasal septum. In 10 (24.39%) patients undergoing ESPAL had blood transfusion whose haemoglobin was less than 8 gm/dl. Most of the patients 36 (87.80%) who underwent ESPAL was discharged on 2nd post-operative day (POD) and only 5 (12.19%) patients were discharged on 1st POD. These patients were followed up 2 weekly for a period of 2 months to see for re-bleed.

## DISCUSSION

Recurrent/intractable epistaxis has high morbidity as well as mortality and its successful management remains a major challenge to an Otorhinolaryngologist. SPA is one of the terminal branches of the internal maxillary artery and the main supply (90%) of the nasal cavity. It has 1-10 branches, and the most anterior branch must be clipped or cauterized and cut, also any other branches if present needs to be identified and cauterized.

Co-morbidities such as hypertension, arteriosclerosis, diabetes, coagulopathy, alcohol and tobacco use, patients on anticoagulants and non-steroidal anti-inflammatory drugs (NSAIDs) are major risk factors for intractable epistaxis in patients. The occurrence of co-morbidities ranges from 30 to 70 % in different studies.^5,6^ This is similar to our study where 31 (75.60%) had co-morbidities with hypertension being the commonest. The age range who underwent ESPAL ranged between 14 to 77 years which was similar to study done by David et al.^7^ Ibrahim et al.^8^, Bijaya K et al.^6^ Prakash A et al.^9^

Male predominance was seen in patients undergoing ESPAL in our study which was similar to study done by Ismi O, et al.^10^ Ibrahim, et al.^8^ Bijaya K et al.^6^ Pramanik and Adhikari^11^ revealed O positive group as the most predominant (35.5%) and AB group as the least prevailing group among Nepalese population which was similar to our study where O positive blood group was found to be predominant 65.85%.

Nasal packing is associated with extreme pain, discomfort, skin, and mucosal necrosis, breathing difficulties, syncope, hypoxia and toxic shock syndrome. So, in recent years, the preference has shifted to endoscopic sphenopalatine artery ligation or cauterization as first-line treatment for posterior epistaxis.^13,14^ Chandler and Serrins first described transantral ligation of the maxillary artery in 1965.^15^ It is highly effective but has significant complications rates. Due to this, the endoscopic transnasal approach for ligation of the sphenopalatine artery has been considered superior with high success rate and no major complications. Over the years, it has replaced the traditional approaches namely internal maxillary artery and external carotid artery ligation. Budrovich and Saetti were the first to report endoscopic ligation of the sphenopalatine artery in 1992.^16^ ESPAL is associated with a shorter hospital stay and is cost-effective compared to other surgical modalities. Endoscopic sphenopalatine artery ligation or cauterization is effective with a success rate ranging from 84-100%.^5,6,14–8^ Our result was similar with 95.12% success rate.

ESPAL failure ranges from 0-16%.^5,13–4,16,19,20^ The reasons as listed by Thakkar et al. include cross anastomosis, dominant contralateral internal maxillary artery, and failure to identify and ligate all branches.^21^ As per O Flynn, et al. multiple branching of the SPA and the variations in the anatomical landmarks could contribute to failure.^5^ Use of aspirin or warfarin, low platelet count on admission also might lead to early failure.^1^ In our study, 2 bleeds within 48 hours. These patients probably had multiple branches of SPA however SPA branches were not made note of in this study. Bilateral ESPAL probably prevented cross anastomosis hence none of them rebled.

This study had some limitations. This was a single centre study and the sample size was small. Due to the small size, this study might not represent generalised to the whole population of Nepal.

## CONCLUSIONS

The findings of the study conclude that ESPAL has a high success rate in patients with intractile posterior epistaxis. From our study, we would like to recommend that endoscopic sphenopalatine artery ligation or cauterization should be preferred as first-line treatment for posterior epistaxis. This study will be beneficial for the development of knowledge by healthcare professionals for the management of posterior epistaxis.
